# Utility of a rapid immunochromatographic strip test in detecting canine parvovirus infection compared with polymerase chain reaction

**DOI:** 10.14202/vetworld.2015.523-526

**Published:** 2015-04-21

**Authors:** Sundaran S. Tinky, R. Ambily, Sreeja R. Nair, Mangattumuruppel Mini

**Affiliations:** Department of Veterinary Microbiology, College of Veterinary and Animal Sciences, Kerala Veterinary and Animal Sciences University, Mannuthy, Thrissur, Kerala

**Keywords:** canine parvoviral diarrhea, immunochromatographic strip test, polymerase chain reaction

## Abstract

**Aim:**

The present study was undertaken to detect the presence of canine parvovirus (CPV) in fecal samples of diarrheic dogs by conventional polymerase chain reaction (PCR) and immunochromatographic (IC) strip test and to compare the diagnostic potential of these tests.

**Materials and Methods:**

A total of 50 fecal samples collected from diarrheic dogs suspected for CPV infection were subjected to PCR using CPV-555 primer amplifying the gene coding for the VP1 protein. These samples were also tested by IC strip test using a commercial rapid Ag test kit. The results were statistically analyzed using McNemar test.

**Results:**

A total of 22 samples (44%) were detected as positive by PCR, which yielded a specific amplicon of 583 bp. In IC strip test, 18 (36%) samples were found to be positive. The sensitivity of the test as compared to PCR was found to be 72.22% and specificity was 92.86%. Positive predictive value and negative predictive value of IC strip test was found to be 88.89% and 81.25%, respectively. Statistical analysis of the results of PCR and IC assay using McNemar test revealed no significant difference (p>0.05).

**Conclusion:**

The IC strip test could be employed as a rapid field level diagnostic tool for the diagnosis of canine parvoviral diarrhea.

## Introduction

Parvoviral enteritis is a highly contagious disease causing heavy mortality in dogs all over the world. Early and rapid diagnosis is quintessential, so that the infected dogs can be isolated and supportive treatment can be adopted to reduce morbidity and mortality [1]. The etiological agent is canine parvovirus (CPV), which emerged in the 1970s as a host range variant of feline panleukopenia virus (FPV). The virus is now classified as Carnivore protoparvovirus 1 [2]. The infection is characterized by acute hemorrhagic gastroenteritis in adult dogs and myocarditis in puppies causing high morbidity and mortality [3].

Diagnosis on the basis of clinical signs is not definitive, since several other pathogenic organisms can cause diarrhea in dogs. Therefore, a clinical diagnosis should always be confirmed with laboratory tests. The standard method for the identification of CPV infection is the detection of morphologically intact virus particles by electron microscopy (EM). This method is fast and specific, but expensive and less sensitive [4]. Isolation of the virus from suspected fecal samples is laborious and time consuming [5]. Thus, although EM and virus isolation are highly specific, they are not often used routinely in a clinical setting [6]. Hemagglutination test seems to be acceptable in routine diagnosis because the test is relatively simple, rapid, and inexpensive, but it is less sensitive and specific [7]. Serological tests fail to diagnose infections in acute stages. Hence, early diagnosis is focused on molecular methods such as polymerase chain reaction (PCR). It is a reliable technique with high degree of sensitivity and specificity in detecting CPV from fecal samples than the conventional antigen or antibody based methods [8,9]. However, the technique needs relatively expensive equipment and reagents, which are not available in routine veterinary practice. This has led to the development of various rapid field level diagnostic test kits based on the principle of immunochromatography [10]. The advantage is that these tests are easy to perform with minimal costs even by the dog owners [11].

However, the efficacies of these rapid tests are often dubious. Considering the above facts, it was decided to detect CPV among diarrheic dogs by PCR and immunochromatographic (IC) strip test and to compare the diagnostic potential of these tests.

## Materials and Methods

### Ethical approval

No ethical approval is necessary for clinical cases. However, all samples were collected as per standard sample collection procedure.

### Collection of sample

Fifty fecal samples were collected from diarrheic dogs in the age group of 2 weeks -8 months of age suspected for canine parvoviral infection that were brought to teaching veterinary clinical complexes of Kerala Veterinary and Animal Science University situated at Mannuthy and Kokkalai. The animals were showing profuse diarrhea with fetid odor and the fecal samples were mixed with blood. The samples were collected from May to August 2014.

The samples for PCR were collected using sterile cotton swabs and were immersed immediately in screw capped polypropylene tubes containing sterile PBS of pH 7.2. For IC strip test, the fecal samples from suspected dogs were collected using sample collection swab, and diluted in one milliliter of assay diluents provided along with the test kit.

### PCR reaction

The template DNA was prepared using crude method of boiling and chilling. Briefly, the fecal samples immersed in PBS were clarified by centrifuging at 9500 ×g for 15 min. at 4°C in a cooling centrifuge (Dynamica). The supernatant was boiled for 10 min. and chilled immediately on ice. It was then centrifuged at 3000 ×g for 10 min. at 4°C and the supernatant was collected into a sterile tube. The concentration and purity of DNA were measured using Nanodrop (Thermo scientific). The DNA was diluted to 1:5 with sterile distilled water, which was then used as template DNA for PCR reaction.

PCR was performed in a volume of 25 µl reaction mixture. The reaction mixture (20 ml) consisted of PCR buffer 1 × (KCl 50 mM, Tris-HCl 100 mM, pH 9), MgCl_2_ 1.5 mM for CPV-555 primer set (These primers were selected from different regions of VP2 gene which codes virus capsid protein. The sequences of primer pairs were as follows: 555 forward, 5’-CAGGAAGATATCCAGAAGGA-3’ and 555 reverse, 5’- GGTGCTAGTTGATATGTAATAAACA- 3’), 200 mM of each deoxynucleotide (dATP, dCTP, dGTP, dTTP), 10 pmol of each primers, 3 U of Taq DNA polymerase, and 5 ml of template DNA. PCR was performed in the thermal cycler (Eppendorf Master cycler gradient TM, Germany) for 30 cycles, each consisting of initial denaturation at 94°C for 5 min, denaturation at 94°C for 30 s, annealing at 55°C for 2 min, extension at 72°C for 2 min, and final extension at 72°C for 5 min. The PCR product obtained was detected by submarine agarose gel electrophoresis.

### IC strip test

It was carried out using a commercial rapid CPV Ag test kit, following the manufacturer’s instructions. The fecal samples collected in assay diluent were left for 10 min for the settlement of larger particles to the bottom. Four drops of supernatant was added into the sample hole. The purple color was observed to move across the result window in the center of the test device. The results were interpreted within 10 min. Control band (C) was located in the left section of the result window. The appearance of a color band was suggestive of proper working of the test. The right section of the result window indicated the test results (T). The development of color band in the right section was taken as positive. The presence of only one band within the result window (C) indicated a negative result. The presence of two color bands (T and C) within the result window indicated a positive result.

### Comparison of PCR and IC strip test

The two tests were analyzed using McNemar test and the results were compared. The sensitivity, specificity, positive likelihood ratio, negative likelihood ratio, positive predictive value, and negative predictive value of IC strip test in comparison with PCR were calculated and interpreted.

## Result

**Figure-1:**
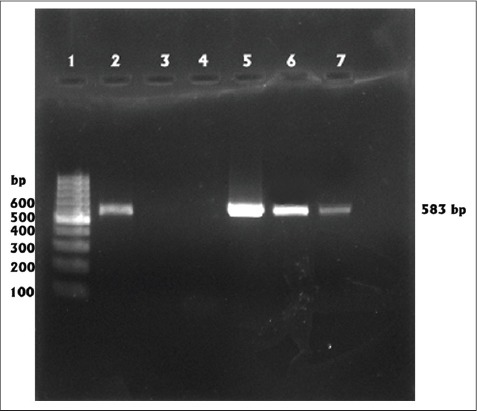
Polymerase Chain Reaction (representation) Lane 1: 100 bp DNA ladder, Lane 5: Positive sample (Megavac P vaccine), Lane 4: Negative control, Lanes 2, 3, 6, 7: samples

**Figure-2:**
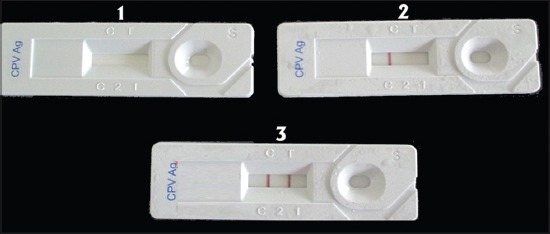
Immunochromatographic strip test (representation) 1. Immunochromatographic strip test device 2. Negative test result 3. Positive test result

An amplicon of 583 bp was detected in 22 out of 50 samples (44%) tested (Figure-1). Among the 50 samples tested using Rapid CPV Ag test kit, 18 (36%) samples were positive (Figure-2). This included two PCR negative samples also. Six samples which were detected as positive in PCR were found to be negative in this test. The tests were compared using McNemar test and no significant difference could be observed between the two tests (p value was calculated as 0.289 which was >0.05). Sensitivity, specificity, positive likelihood ratio, negative likelihood ratio, and negative predictive values of IC strip test in comparison with PCR were analyzed (Table-1). The sensitivity of IC strip test was found to be 72.22% and specificity was 92.86%.

**Table-1:** Comparison of PCR and IC strip test.

	PCR positive	PCR negative
IC positive	16	2
IC negative	6	26

PCR=Polymerase chain reaction, IC=Immunochromatographic strip test

Sensitivity=72.73%
Specificity=92.86%
Positive likelihood ratio=10.18
Negative likelihood ratio=0.29
Disease prevalence=44.00%
Positive predictive value=88.89%
Negative predictive value=81.25%

## Discussion

Parvoviral infections in dogs have become an important problem globally. The clinical signs resemble other enteric diseases and hence rapid and early diagnosis of the condition has become ever more urgent. Conventional methods such as EM and virus isolation are time-consuming, less sensitive, and expensive [4-6]. Serological tests could detect the antibody, but fail to detect the acute infection. The widely used HA HI tests are simple, but are less sensitive and always require supply of fresh erythrocytes [5,7]. Hence, these tests are now replaced by molecular methods like PCR which is having high specificity [12] and sensitivity [13] than the conventional antigen or antibody-based methods. However, the necessity of expensive equipment and reagents restricts its use as a field level test [9]. The most rapid method for diagnosing parvoviral infections in practice is IC based canine fecal antigen test kits which are sensitive, simple, and rapid [11]. The test is easy to perform [6] even by owners [4] without any specialized equipment [9].

In the present study, of the 50 samples subjected to IC strip test, 18 (36%) were detected as positive for CPV infection whereas 22 (44%) were positive by PCR. Two samples which were observed to be positive in IC strip test, failed to give a positive result in PCR. The presence of inhibitory substances in feces might have interfered with PCR assay. This is in accordance with previous studies [14] who reported false negative results in PCR due to inhibitory substances in the feces.

Six PCR positive samples failed to give a positive result in IC strip test. This may be due to the requirement of large amount of viral antigen to produce a clearly visible band. This result is in agreement with others, [9] who reported that the quantity of viral particles can affect the IC test result which was observed to be one of the disadvantages of this test. It is proved that samples with viral load more than 109 DNA copies/mg feces were generally detected by in-house assay [15]. There were previous reports [16] stating that rapid antigen detection tests have high specificity but poor sensitivity when compared to PCR. However, when compared to the HI assay, canine fecal antigen test kit had 97.1% sensitivity, and 76.6% specificity [17]. Quick test kits can detect the presence of parvovirus, but will not be able to distinguish the type involved [18].

In the present study, sensitivity and specificity of IC strip test compared to PCR was found to be 72.73% and 92.86%, respectively. The results are in accordance with previous studies [11] which stated PCR as a more sensitive test than IC strip test. However, several others [4] noticed that the IC assay were found to be highly specific (98.8%) and sensitive (100%). Certain researchers [9] reported that the sensitivity of IC was 84% in PCR-positive samples. In the present study, a positive predictive value of 88.89% and negative predictive value of 81.25% could be observed. The high positive likelihood ratio (10.18) and low negative likelihood ratio (0.29) also suggests the efficacy of IC as a reliable diagnostic test for the detection CPV antigen in fecal samples. Moreover, the statistical analysis of the results of PCR and IC using McNemar test revealed no significant difference. Hence, IC strip test could be recommended as a rapid field level test for diagnosing CPV infections in routine veterinary practice.

## Conclusion

This study shows that the IC strip test could be recommended as a rapid field level diagnostic tool for the diagnosis of CPV infections in dogs.

## Authors’ Contributions

SST conducted the research under the guidance of RA. SRN and MM were involved in scientific discussion and analysis of the result of the research work. All authors participated in draft and revision of the manuscript. All authors read and approved the final manuscript.
